# Genetic evidence for imported malaria and local transmission in Richard Toll, Senegal

**DOI:** 10.1186/s12936-020-03346-x

**Published:** 2020-08-03

**Authors:** Rachel F. Daniels, Stephen F. Schaffner, Yakou Dieye, Gnagna Dieng, Michael Hainsworth, Fatou B. Fall, Coumba Ndoffene Diouf, Medoune Ndiop, Moustapha Cisse, Alioune Badara Gueye, Oumar Sarr, Philippe Guinot, Awa B. Deme, Amy K. Bei, Mouhamad Sy, Julie Thwing, Bronwyn MacInnis, Duncan Earle, Caterina Guinovart, Doudou Sene, Daniel L. Hartl, Daouda Ndiaye, Richard W. Steketee, Dyann F. Wirth, Sarah K. Volkman

**Affiliations:** 1 Harvard T.H. Chan School of Public Health, Boston, MA USA; 2 Broad Institute, Cambridge, MA USA; 3 PATH MACEPA, Seattle, WA USA; 4 Senegal National Malaria Control Programme, Dakar, Senegal; 5 District Medical Office, Richard Toll District, Richard Toll, Senegal; 6 Dantec Teaching and Research Hospital, Dakar, Senegal; 7 Centers for Disease Control and Prevention, Atlanta, GA USA; 8 Harvard University, Cambridge, MA USA; 9 Cheikh Anta Diop University, Dakar, Senegal; 10 Simmons University, Boston, MA USA

**Keywords:** Molecular epidemiology, Imported, Local transmission, Malaria surveillance, Malaria pre-elimination

## Abstract

**Background:**

Malaria elimination efforts can be undermined by imported malaria infections. Imported infections are classified based on travel history.

**Methods:**

A genetic strategy was applied to better understand the contribution of imported infections and to test for local transmission in the very low prevalence region of Richard Toll, Senegal.

**Results:**

Genetic relatedness analysis, based upon molecular barcode genotyping data derived from diagnostic material, provided evidence for both imported infections and ongoing local transmission in Richard Toll. Evidence for imported malaria included finding that a large proportion of Richard Toll parasites were genetically related to parasites from Thiès, Senegal, a region of moderate transmission with extensive available genotyping data. Evidence for ongoing local transmission included finding parasites of identical genotype that persisted across multiple transmission seasons as well as enrichment of highly related infections within the households of non-travellers compared to travellers.

**Conclusions:**

These data indicate that, while a large number of infections may have been imported, there remains ongoing local malaria transmission in Richard Toll. These proof-of-concept findings underscore the value of genetic data to identify parasite relatedness and patterns of transmission to inform optimal intervention selection and placement.

## Background

Senegal is characterized by distinct malaria transmission zones, ranging from very low in the North (incidence < 1/1000) to moderately high in the South (incidence > 400/1000). Richard Toll, a northern district, reports one of the lowest incidence levels in Senegal (0.2/1000 persons in 2017) [1]. Only a few hundred malaria cases are detected in Richard Toll each year. The majority of confirmed cases have recent travel history, and thus are classified as imported malaria [2]. Much of this travel is related to seasonal migrant workers at the Senegalese Sugar Company, who come from locations outside Richard Toll where malaria transmission levels are higher [2]. Travel history for classification of imported malaria may be compromised by a number of factors including recall bias, uncertainty about infection origin, and underreporting of imported infections [3–5]. Thus, improved methods for assessing the risk of importation are needed.

Understanding the risk and pattern of malaria transmission in a given geographical area provides the foundation for intervention selection and targeting to decrease malaria burden, eliminate transmission, and prevent re-establishment of malaria. The risk of malaria transmission is the product of the risk of local spread, rate of importation, and infectivity, and is referred to as the *malariogenic* potential [6]. The World Health Organization (WHO) recommends that these parameters inform the selection and targeting of interventions for eliminating malaria transmission [7]. Thus, metrics that better identify and quantify the rate of importation and risk of local spread of malaria infections are required for successful malaria elimination.

Population genetic signatures may reflect changes in transmission dynamics [8] and can imply genetic relatedness between parasites [9, 10]. Strategies that quantify the proportion of the genome that is identical between two parasites by being derived from a common ancestor (i.e., identical by descent) [11] may reveal changes in transmission [8], resolve multi-genome infections [12], facilitate characterization of drug-resistance markers [13], detect signals of selection [14], and create fine-scale maps of parasite dispersion based upon geographic distance [10]. Simple genotyping tools, including the molecular barcode that interrogates 24 single nucleotide polymorphisms (SNPs) [15], reveal patterns of parasite relatedness including the persistence of genetically identical parasite lineages from one transmission season to the next [8, 16]. More complex genotype infections, defined as the presence of more than one parasite genome in a single patient, are found in higher transmission settings as a consequence of outcrossing between genetically distinct parasite types [17]. Thus, detection of more complex parasite genotypes among travellers in low transmission settings is consistent with these infections originating from higher transmission areas [18–23]. Genetic data can provide more precise information about infection origin and connectivity (i.e., the degree of genetic relatedness) between infections [8], information that can be used to determine if a malaria infection is local or imported.

To test the hypothesis that genetic relatedness can be used to differentiate imported and local infections, this pilot project applied population genetic analysis to samples collected during malaria elimination efforts in Richard Toll, Senegal. These activities included case investigation of all passively identified malaria infections (index cases) with reactive active case detection targeting the household and neighbouring households of the index case between 2012 and 2015 [2]. Malaria-positive samples from this case-investigation were subjected to genetic relatedness analysis to test the hypothesis that most if not all Richard Toll infections were imported.

## Methods

### Sampling scheme

Routine case investigation was carried out in Richard Toll, Senegal between September 2012 and December 2015 through a partnership with the Senegal National Malaria Control Programme (Programme National de Lutte contre le Paludisme, PNLP), PATH’s Malaria Control and Elimination Partnership in Africa (MACEPA), and local health facility and community health workers [2]. Rapid diagnostic tests (RDTs) used to diagnose malaria cases detected through facility-based passive case detection (PCD) or during reactive case detection (RACD) were used to genotype all malaria infections. All individuals with RDT-positive malaria infection diagnosed at a health facility (index cases) were followed up within 3 days by a community team that conducted detailed case investigation with RACD that involved visiting the index case household, where all individuals at least 6 months of age were tested by RDT, and the five closest neighbouring households. In the neighbouring households, the RACD strategy was changed from testing all individuals in 2012–2014 to testing only those with at least one risk factor (travel history, reported fever in previous 7 days and/or not sleeping under a bed net) in 2015. This change was based upon the fact that only 1 infection was identified in a neighbouring house in 2014, and for comparison there were 2 infections identified in neighbouring houses in 2015, indicating that this sampling strategy did not likely change the comparison between years. All RDT-positive individuals found during the RACD were treated with Coartem^®^. A standardized questionnaire was filled out for all participants to collect information on basic demographic information including travel history, with a traveller defined as a resident who spent at least one night outside Richard Toll district in the previous 14 days. A total of 759 discarded positive RDT samples were obtained from either index cases (*n* = 673) or secondary infections found during RACD (*n* = 86) between 2012 and 2015 in Richard Toll district.

### Genotyping

Nucleic acid material was extracted from the RDT and pre-amplification [24] with molecular barcode genotyping was performed as previously described [15]. Briefly, the filter pad from the RDT was used to extract genomic DNA using the manufacturer protocol from Promega Maxwell DNA IQ Casework Sample kit (Promega AS1210). Samples were subjected to pre-amplification, then molecular barcode analysis.

### Allele calling

Genotypes were called by their base designation for DNA: A, T, G, or C. Missing alleles are indicated by an “X”; and ascertained alleles that are called bi-allelic (i.e., both the minor and major allele are called by genotyping) are designated by an “N”.

### Genotyping quality control

Any 24-SNP barcode that was missing 4 or fewer assays was included in the analysis and considered as successfully genotyped. This means that barcodes missing 0, 1, 2, 3, or 4 assays were included in the analysed samples. Any sample missing 5 or more assays of the 24 SNPs in the barcode were regarded as unsuccessfully genotyped.

### Data analysis

This study included all samples that passed genotyping quality control for calculating the proportion of infections that were monogenomic (see definitions, Table 1). Only monogenomic infections were included for the pairwise relatedness comparisons. Pairwise analysis was performed to estimate identity by state (IBS) using the software package hmmIBD [9]. Default parameters were used except that the maximum number of iterations was doubled to ten and barcode assays were treated as essentially unlinked by invoking a large genetic distance. IBS analysis was used given the small number of loci in the barcode. Previous analysis has shown that IBS is a good proxy for identity by descent (IBD) and that parasites with barcode sequences with > 0.95 relatedness are genetically identical by sequencing analysis [8]. The IBS estimate used in the software output was “fract_sites_IBD.” Only sample pairs that were identical or highly related (definitions, Table 1) were included in subsequent analyses, which corresponded to a value of 0.95 or greater [9]. This corresponded to a minimum inclusion criterion of 22/23 barcode positions matching between two parasites (Additional file 1).

**Table 1:** Definitions

Term	Definitions for successfully genotyped samples
Monogenomic	0 or 1 N calls
Polygenomic	2 or more N calls
Identical	24/24 or 23/23 matched calls
Highly related	23/24 or 22/23 matched calls
Barcode group	Parasites with pairwise relatedness of IBS > 0.95
Lineage	Parasites with pairwise relatedness of IBS > 0.95 detected in multiple years (transmission seasons)

### Statistical analysis

The hypothesis that the proportion monogenomic and polygenomic (definitions, Table 1) was significantly different between travellers and non-travellers was assessed using a one-tailed Fisher Exact Test for small sample sizes, with a significance level of *α* = 0.05 (Mathematica 10.4.0). The hypothesis that the means were significantly different between household sample sets from travellers and non-travellers was assessed using a two-tailed Fisher Exact Test for small sample sizes, with a significance level of *α* = 0.05 (Mathematica 10.4.0).

## Results

### Polygenomic infections are more common among travellers to Richard Toll

**Fig. 1:**
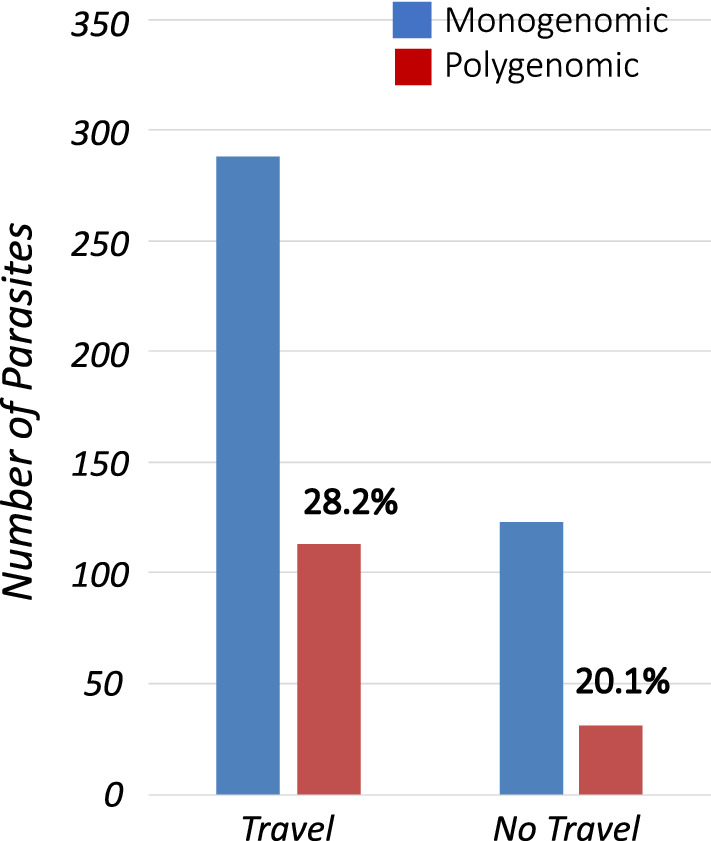
Increased likelihood of polygenomic samples among individuals with recent travel history. Comparison of samples (n = 555) that were successfully genotyped with travel history stratified by genotype (monogenomic *vs*. polygenomic) and recent travel or no travel reported (travel vs. no travel). A significant enrichment of polygenomic infections (Fisher Exact Test, one-tailed, p = 0.03) was detected among individuals with travel history (28.2%) compared to similar infections among individuals with no travel history (20.1%). See Additional file 2 for raw numbers and more details

To determine whether malaria infections obtained from travellers and non-travellers exhibited different levels of genetic diversity, the genotyping data were analysed from all samples obtained from individuals who provided travel history information. Among infected individuals providing travel history information, 72% (401/555) indicated that they had travelled and spent at least one night outside Richard Toll district in the previous 14 days (Additional file 2). Genotyping data from infections derived from travellers were then compared to genotyping data from non-traveller malaria infections. Evaluation of the number of monogenomic and polygenomic infections for each of these two populations (travellers vs. non-travellers) revealed a significant increase in number of polygenomic infections among individuals who reported recent travel outside Richard Toll (28.2%), compared to infections identified in non-travellers (20.1%) (one-tailed Fisher’s Exact Test, *P* = 0.032, Fig. 1, Additional file 2).

### A large proportion of Richard Toll parasites are genetically related to parasites from Thiès

**Fig. 2:**
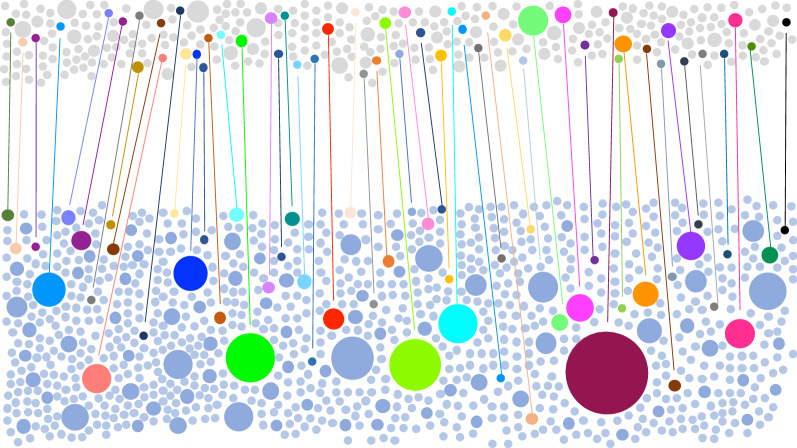
Shared parasite genotypes between Richard Toll and Thiès. Pairwise comparisons of *Plasmodium falciparum* genotype data from Richard Toll (n = 426 in upper panel) and Thiès (n = 1516 in lower panel) reveal that 88 of 426 (21%) of Richard Toll parasites are highly related to one of 343 Thiès parasites and comprise 52 barcode groups represented by coloured circles connected by lines to represent individual parasite genotypes that are shared across populations. The coloured circles are area-scaled based upon the number of infections per genotype. Individual (unique) Richard Toll parasites are shown in grey at the top and Thiès parasites in blue at the bottom. The number of samples that are unique (240 Richard Toll and 869 Thiès) represent barcodes that are not highly related to any other parasite. There are 35 barcode groups comprising highly-related parasites from Richard Toll only (n = 98 parasites) and 75 barcode groups that contain highly related parasites from Thiès only (n = 869). These are represented as individual grey or blue circles, respectively

To test whether malaria parasites detected in Richard Toll matched parasites from elsewhere in Senegal, genotyping data from Richard Toll parasites were compared to a large existing genotyping database of Senegal parasites isolated from Thiès, a region of higher malaria transmission that has undergone dramatic declines in malaria burden since 2001. Pairwise analysis of relatedness, including identity by state (IBS), was performed on genotyping data from both Richard Toll samples and clinical samples collected during research conducted in Thiès. A total of 426 Richard Toll samples and 1,516 Thiès samples met the criteria of being monogenomic and missing either no or one single nucleotide polymorphism (SNP) assay (Additional files 3, 4). While the Thiès sample set represents only a fraction of malaria infections in Senegal, it provided pilot data for this proof of concept study. Genotype data were evaluated for relatedness by calculating the proportion of alleles that matched between pairs of samples to identify both identical and highly related sample sets (definitions, Table 1) (Additional file 5). This pairwise analysis revealed that a large proportion (21%) of Richard Toll infections (Additional file 3) shared an identical or highly related barcode with parasites from Thiès (Additional file 4), Senegal (Fig. 2, Additional files 3, 4). For comparison, any two other populations within Senegal in the same time frame only share a small proportion of infections (< 5%) sampled.

### Persistence of genetically identical infections across multiple transmission seasons

**Fig. 3:**
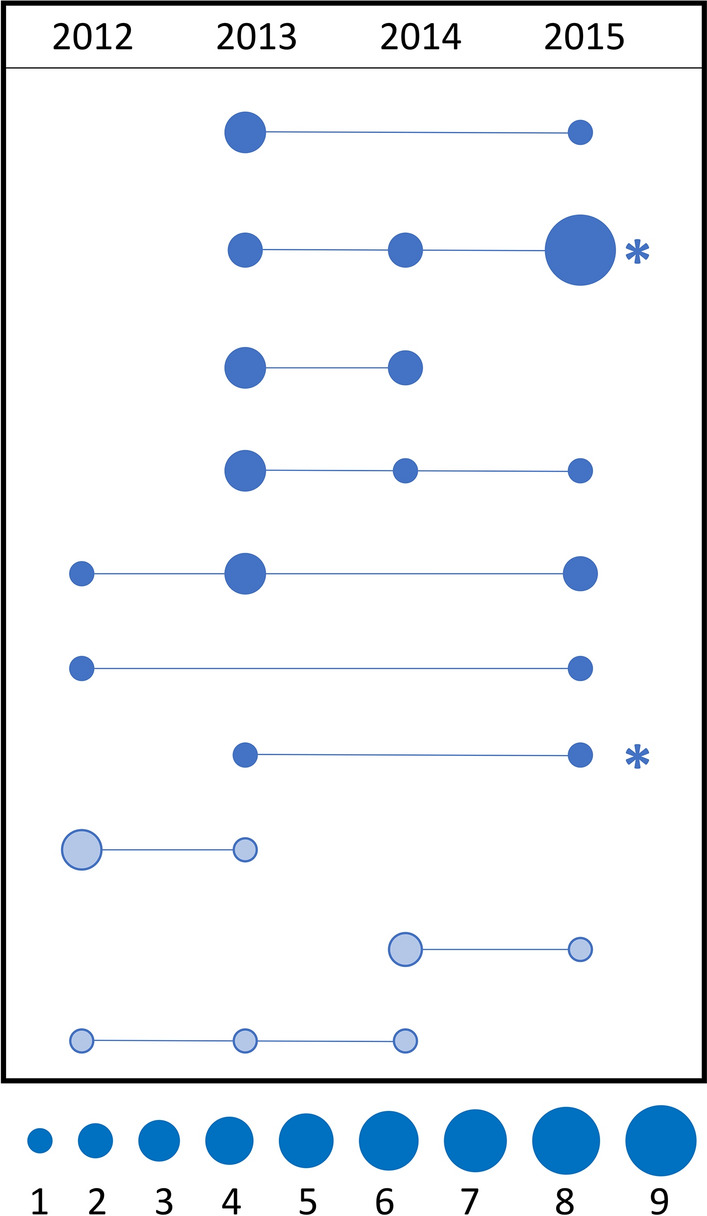
Richard Toll parasites with identical or highly related genotypes are detected in multiple years. Stick and ball schematic representing the number of Richard Toll parasite lineages [identical (n = 7; solid blue) or highly related (n = 3; striped blue)] or genotypes that persist for multiple years between 2012 and 2015. The size of the circle (area scaled) represents the number of parasites, from 1 parasite (smallest) to 9 parasites (largest), with a line connecting these parasite groups across years. Lineages with an asterisk (*) represent the two parasite genotypes that matched a parasite from Thiès

To ask whether genetically identical parasites from Richard Toll were detected in multiple transmission seasons, barcode groups identified by the pairwise relatedness analysis were examined (definitions, Table 1). Transmission seasons in Senegal are seasonal and generally occur between September and December of the calendar year in northern and central Senegal. A barcode group contained identical or highly related genotypes from the pairwise analysis, and the genotype associated with barcode groups that contained parasites from multiple years represent a parasite lineage (definition, Table 1). This analysis revealed seven genetically identical parasite lineages and three highly related parasite lineages, with these genotypes found in more than one transmission season between 2012 and 2015 (Fig. 3, Additional file 6). Two of these barcode groups representing lineages contained parasites identical or highly related to genotypes from Thiès, while the remaining lineages contained only parasites detected in Richard Toll. In one of these, the genotype appeared in two cases in 2013, again in two cases in 2014, and expanded to nine cases in 2015.

### Highly related parasites found in households of non-travellers

**Fig. 4:**
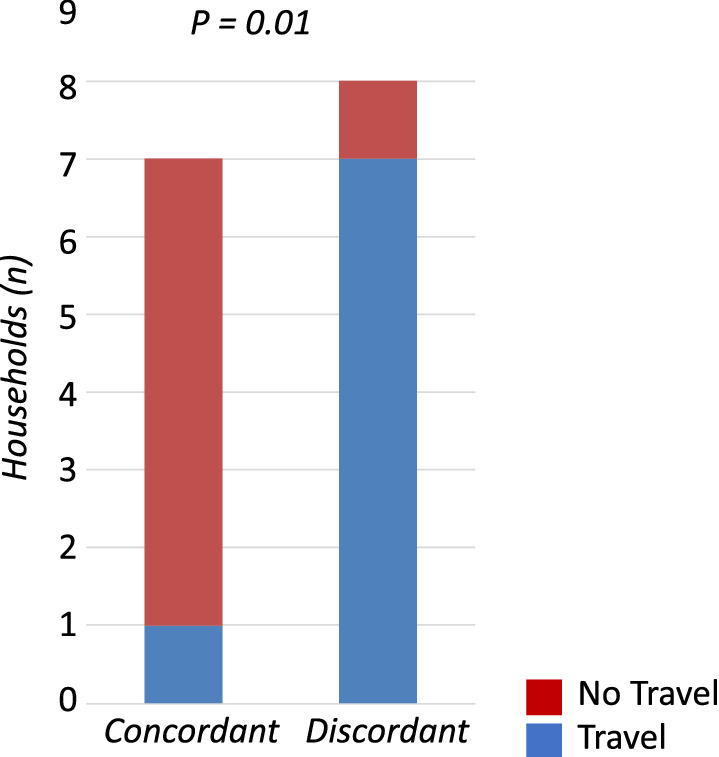
Highly related parasites found within households with no travel history. Genotypes of parasites from the index case detected passively at clinic and any household members detected actively in the household during follow-up were compared and scored for relatedness. Highly related barcode groups were identified and distributed based upon whether or not infections were found in travellers. A total of 15 households had infections actively detected, with 7 of these households reporting no travel history and the remaining 8 households reporting recent travel. Highly related parasites were more likely (*p *=* 0.01*) to be found within households with no travel history (6/7) compared to households with travel history (1/8). Data by year are shown in Additional file 7

To ask whether infections from travellers contribute to a risk of local spread of these imported infections, this study took advantage of the RACD programme, in which all household members received an RDT, [2] and tested whether parasites within households share genetic relatedness. The index case’s infection was detected in the clinic, and the additional infections were found among household members of the index case. Only fifteen households (of 49 households in which RACD was conducted and genotyping data was available) had both an initial infection and at least one additional infection that yielded genotyping data for the comparison. These within-household infections were scored for genetic relatedness using pairwise analysis, then households that harboured highly related infections were classified according to whether the index case had travel history. These households were first scored based upon whether there was evidence of highly related infections and found that about half (7/15, 47%) of these households had at least one additional infection that genetically matched (i.e., identical or highly related) the initial infection (Fig. 4, Additional files 7, 8). Of seven households with at least one infected person in which there was no travel history, six contained at least two identical or highly related parasites, while of eight households with at least one infected person and travel history, only one household contained at least two identical or highly related parasites. Stratification of households based upon travel history revealed a significant increase of highly related parasites within households with no travel history (*P* = 0.01).

## Discussion

Richard Toll represents a region with one of the lowest levels of malaria incidence in Senegal. Most malaria cases in Richard Toll are detected in individuals who had travelled in the previous 2-week period, thus are classified as imported infections [2]. A genetic epidemiological approach was applied to compare the relatedness of malaria infections within Richard Toll as well as between Richard Toll and Thiès, Senegal in a proof of concept study. First, it was observed that polygenomic infections are more common among travellers to Richard Toll, consistent with the hypothesis that these represent infections imported from higher malaria transmission settings outside Richard Toll. Pairwise analysis [9] of genotypes from 649 Richard Toll infections and 1555 Thiès infections revealed that 21% of Richard Toll samples genetically matched parasites from Thiès, consistent with importation of Richard Toll infections. A substantially greater proportion of malaria cases diagnosed in Richard Toll are likely to be imported; while Thiés is Senegal’s third largest city, travel to Thiès accounts for a relatively small proportion of reported travel history [2].

However, genetic relatedness among infections from within Richard Toll revealed patterns of ongoing local transmission. Evidence for local transmission includes, first, that multiple genetically identical parasite lineages found across multiple transmission seasons were detected in Richard Toll; and second, that highly related infections were more likely to be found within households that lacked recent travel. While the sample numbers in this study are small, both of these findings are consistent with local and focal transmission. Given that only one household was found both multiple identical genotypes and a travel history suggests that local transmission of an imported malaria case may be a relatively rare event.

Successful malaria elimination benefits from identifying the origin and means of transmission for the few remaining cases in regions of very low transmission. It is important to determine the rate of importation and the risk of local spread in these regions approaching elimination [7]. The WHO recommends that information related to the rate of importation and risk of local spread be used for optimal targeting and stratification of interventions to achieve elimination success [7]. Importation can lead to introduced cases, which can persist for multiple transmission seasons as local, indigenous cases. The majority of the passive case infections were found among individuals with a recent travel history. Based upon the observation that most infections are among travellers, there has been an assumption of no, or very limited, local transmission. Assumptions that most or all malaria infections are the result of importation can impact operational activities such as rather than testing all fevers for malaria, testing fevers among travellers; or a reduction or abandonment or prioritization of using vector control interventions in the district.

## Conclusions

This pilot activity demonstrates the feasibility and likely success of genetic approaches for mapping potential transmission patterns related to imported and local infections. Samples from other sites and additional genetic data, such as obtained from sequencing, will improve resolution and understanding of gene flow between geographic regions. In addition to improvements in sequencing parasite genomes directly from clinical samples, development of population genetic strategies, including analysis of identity by descent, will help define the relationships between infections in settings approaching malaria elimination. The use of identity by descent may help delineate relationships between parasite infections through quantification of the proportion of the genome shared between two parasites derived from a common ancestor, as well as the length of these shared genomic tracts, to estimate the number of generations between individual parasite infections. Challenges of how to address polygenomic infections remain, and may reveal important patterns of transmission, particularly in regions of high malaria burden. Incorporation of other data including human mobility with genetics and modelling these data will also improve our ability to evaluate the impact of imported malaria infections to ongoing transmission of malaria [25]. These and other population genetic approaches provide important information for mapping the rate of importation and risk of local transmission in specific geographic regions to reduce the malaria burden. This work underscores the value of and the opportunity for genetic data to reveal patterns of transmission that can help inform optimal intervention selection and placement for successful malaria elimination [26]. The findings of the present study demonstrate the usefulness of genetic tools to inform interventions to eliminate local transmission and prevent further introduction of imported malaria parasites that may contribute to local transmission.

## Supplementary information

**Additional file 1.** Schematic of Richard Toll sample analysis. A total of 759 samples were received and genotyping yielded 649 samples that passed genotyping (5 or fewer missing alleles in the 24-SNP barcode). Of these 649 samples, 473 were monogenomic (0, or 1 mixed allele in the 24-SNP barcode).

**Additional file 2.** Travel history and genotyping data.

**Additional file 3.** Richard toll parasite genotyping data.

**Additional file 4.** Thiès parasite genotyping data.

**Additional file 5.** Pairwise comparison of genotyping data using identity by state (IBS) analysis.

**Additional file 6.** Persistent parasite lineages.

**Additional file 7.** Household comparisons.

**Additional file 8.** Map of infections. A. Map of infections collected in 2015 and relative location of Thiès. The distance between Richard Toll and Thiès is approximately 150 miles. The sites of households from which samples were collected in 2015 are shown, with red indicating infections in individuals with a travel history and blue indicating infections from individuals with no travel history (see B). No household locations were collected in any other year. B. Inset from panel A is shown enlarged to indicate the relative locations of households from which parasite infections were identified in 2015. Infections identified in individuals who reported a travel history are shown in red, while those from individuals who reported no recent travel are shown in blue. Tear drop markers indicate infections identified through reactive active case detection (RACD), with index case marked red for positive travel history or blue for no travel history. All of these RACD households with a travel history (red teardrops) had parasites that were different from one another; and all these RACD households with no travel history (blue teardrops) shared genotype infections, with one exception being the household in dark blue in the center of Richard Toll (identified with an asterisk). This household had parasites that were genetically distinct but the index case reported no recent travel. C. Location of passive case detection infections that share a parasite genotype. To provide an example of household location where shared infections were identified by passive case detection at four households with the same parasite genotype are shown in blue teardrops, and a different parasite genotype found among two households are shown in red teardrops. The two infections from the red household were both detected on the same date at the clinic. The infections for the blue households occurred on different dates: (1) September 7; (2) September 12; (3) November 8; (4) November 10. Note that household 4 was positive for additional infections and corresponds to the household in panel B as indicated by the red arrow. The geographic distance between household 1 and 2 is 3.3 miles; between household 2 and 3 is 14 miles and between household 3 and 4 is 0.8 miles.

## Data Availability

The dataset(s) supporting the conclusions of this article is(are) included within the article (and its additional file(s)).

## Abbreviations

IBD: Identity by descent
IBS: Identity by state
MACEPA: Malaria Control and Elimination Partnership in Africa
PCD: Passive case detection
PNLP: Programme National de Lutte contre le Paludisme
RACD: Reactive active case detection
RDT: Rapid diagnostic test
SNP: Single nucleotide polymorphism
WHO: World Health Organization
