# Supplementary material for: Genetic evidence for imported malaria and local transmission in Richard Toll, Senegal
Source: Malar J. 2020 Aug 3;19:276. doi: 10.1186/s12936-020-03346-x (PMC7397603; doi:10.1186/s12936-020-03346-x)
Supplement: Supplementary file 1 — Additional file 1. Schematic of Richard Toll sample analysis. A total of 759 samples were received and genotyping yielded 649 samples that passed genotyping (5 or fewer missing alleles in the 24-SNP barcode). Of these 649 samples, 473 were monogenomic (0, or 1 mixed allele in the 24-SNP barcode). [file 12936_2020_3346_MOESM1_ESM.pdf]

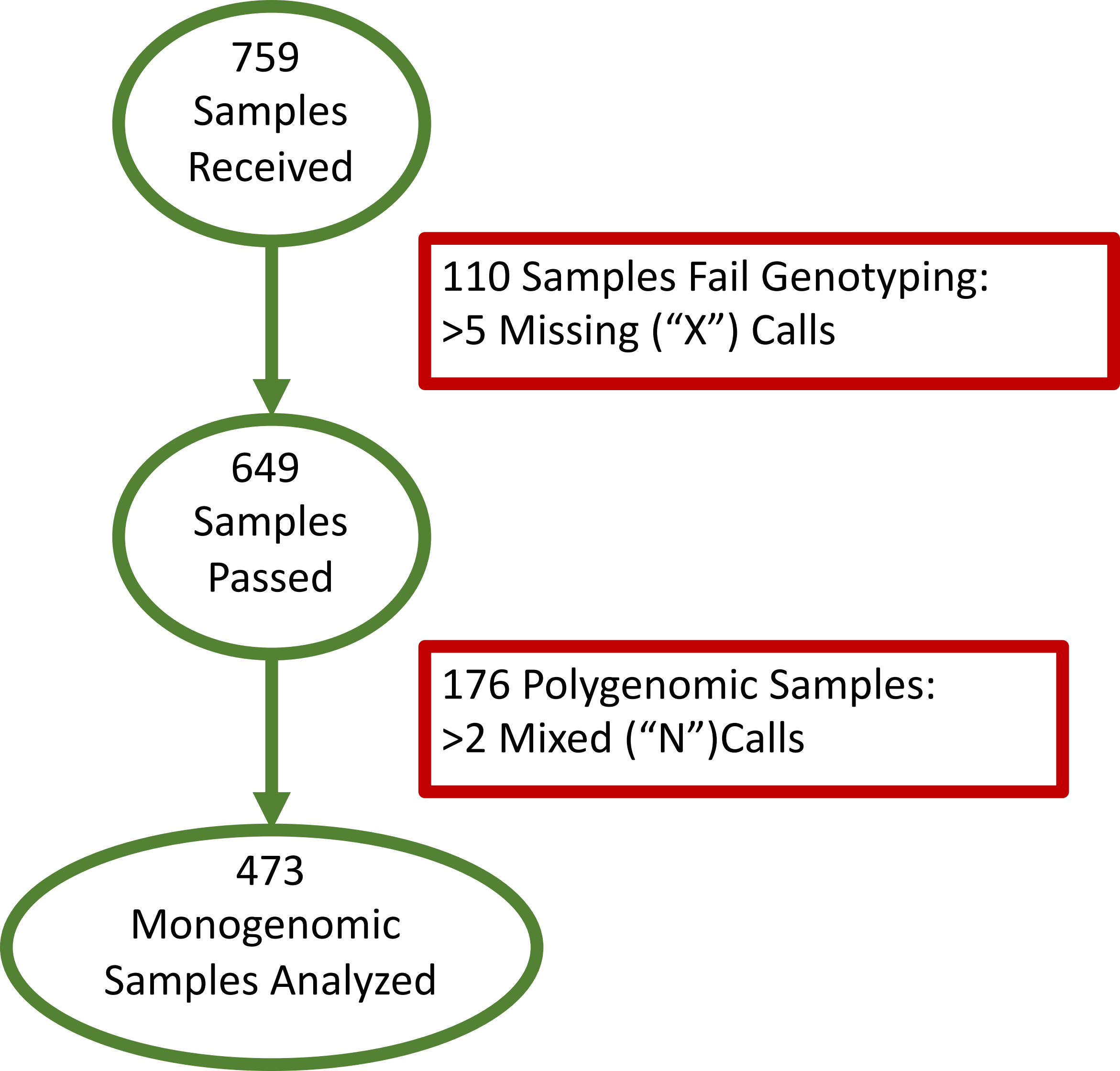
759
Samples
Received
110 Samples Fail Genotyping:
>5 Missing (“X”) Calls
649
Samples
Passed
176 Polygenomic Samples:
>2 Mixed (“N”)Calls
473
Monogenomic
Samples Analyzed
