# Supplementary figures and images for: Genetic evidence for imported malaria and local transmission in Richard Toll, Senegal
Source: Malar J. 2020 Aug 3;19:276. doi: 10.1186/s12936-020-03346-x (PMC7397603; doi:10.1186/s12936-020-03346-x)

## Additional File 8: Map of Samples from 2015

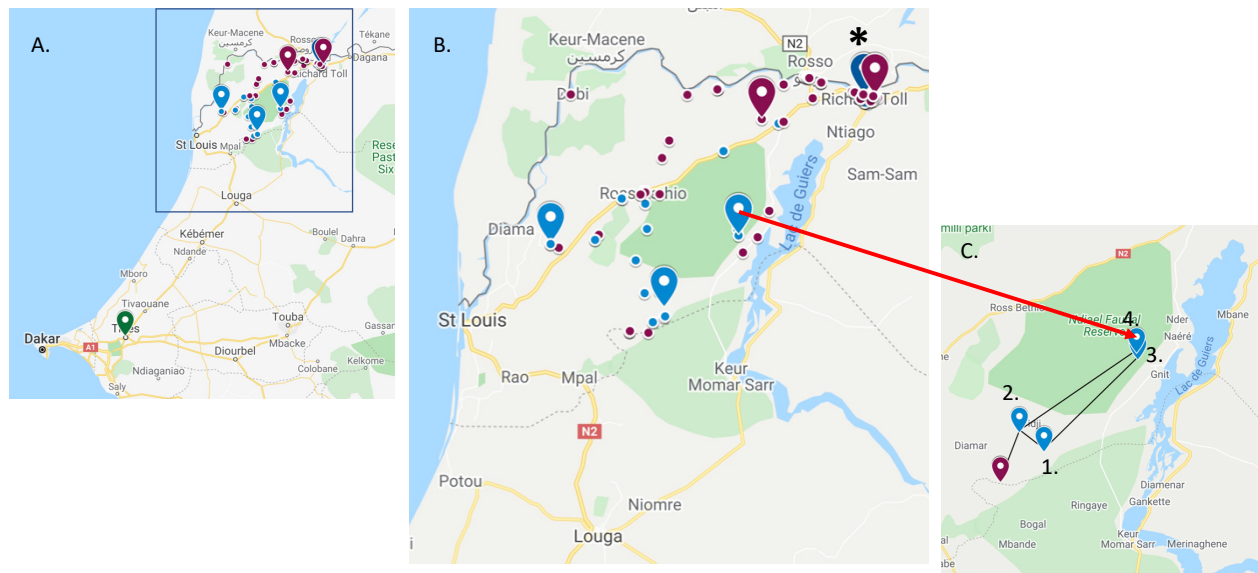

Supplement: Supplementary file 8 — Additional file 8. Map of infections. A. Map of infections collected in 2015 and relative location of Thiès. The distance between Richard Toll and Thiès is approximately 150 miles. The sites of households from which samples were collected in 2015 are shown, with red indicating infections in individuals with a travel history and blue indicating infections from individuals with no travel history (see B). No household locations were collected in any other year. B. Inset from panel A is shown enlarged to indicate the relative locations of households from which parasite infections were identified in 2015. Infections identified in individuals who reported a travel history are shown in red, while those from individuals who reported no recent travel are shown in blue. Tear drop markers indicate infections identified through reactive active case detection (RACD), with index case marked red for positive travel history or blue for no travel history. All of these RACD households with a travel history (red teardrops) had parasites that were different from one another; and all these RACD households with no travel history (blue teardrops) shared genotype infections, with one exception being the household in dark blue in the center of Richard Toll (identified with an asterisk). This household had parasites that were genetically distinct but the index case reported no recent travel. C. Location of passive case detection infections that share a parasite genotype. To provide an example of household location where shared infections were identified by passive case detection at four households with the same parasite genotype are shown in blue teardrops, and a different parasite genotype found among two households are shown in red teardrops. The two infections from the red household were both detected on the same date at the clinic. The infections for the blue households occurred on different dates: (1) September 7; (2) September 12; (3) November 8; (4) November 10. Note that household 4 [file 12936_2020_3346_MOESM8_ESM.pdf]
